# Supplementary material for: Standardization of Data Analysis for RT-QuIC-Based Detection of Chronic Wasting Disease
Source: Pathogens. 2023 Feb 13;12(2):309. doi: 10.3390/pathogens12020309 (PMC9962701; doi:10.3390/pathogens12020309)
Supplement: Supplementary file 1 [file pathogens-12-00309-s001.zip › pathogens-2075257-supplementary.pdf]

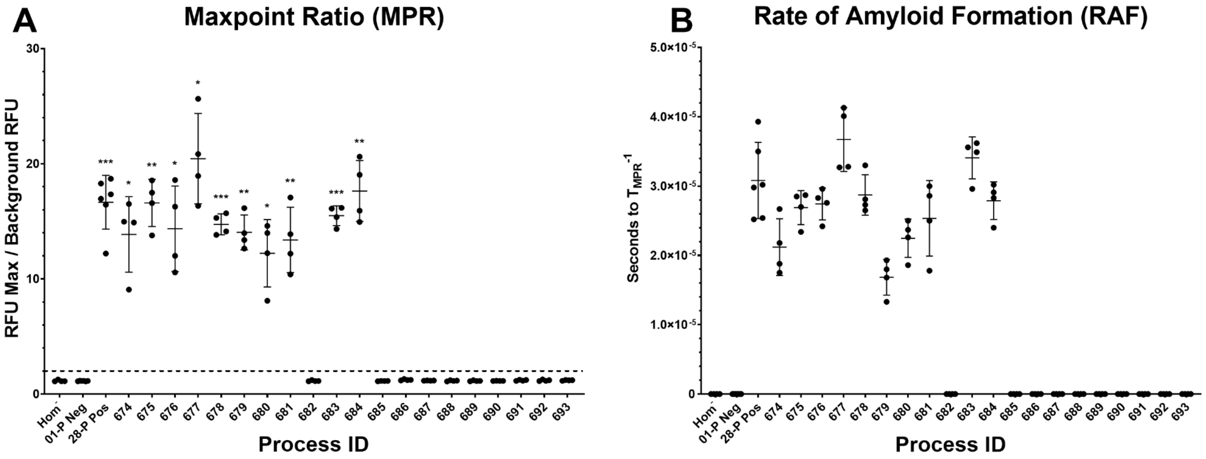

Supplementary Figure S1 - Example graphs highlighting the maxpoint ratio (MPR) and their correlated amyloid formation rates (RAF). (**A**) MPRs for each sample are shown with bars at the median extending one standard deviation. The dotted line represents the MPR threshold (T_{MPR}) for RAF calculation and is set permanently at two. Significance is determined by comparing samples' MPRs to the negative control MPRs with a one-way ANOVA and a Dunnett's multiple comparisons test (*, p>0.05; **, p>0.01; ***, p>0.001). (**B**) RAFs were calculated by taking the reciprocal of seconds needed to pass T_{MPR}.
